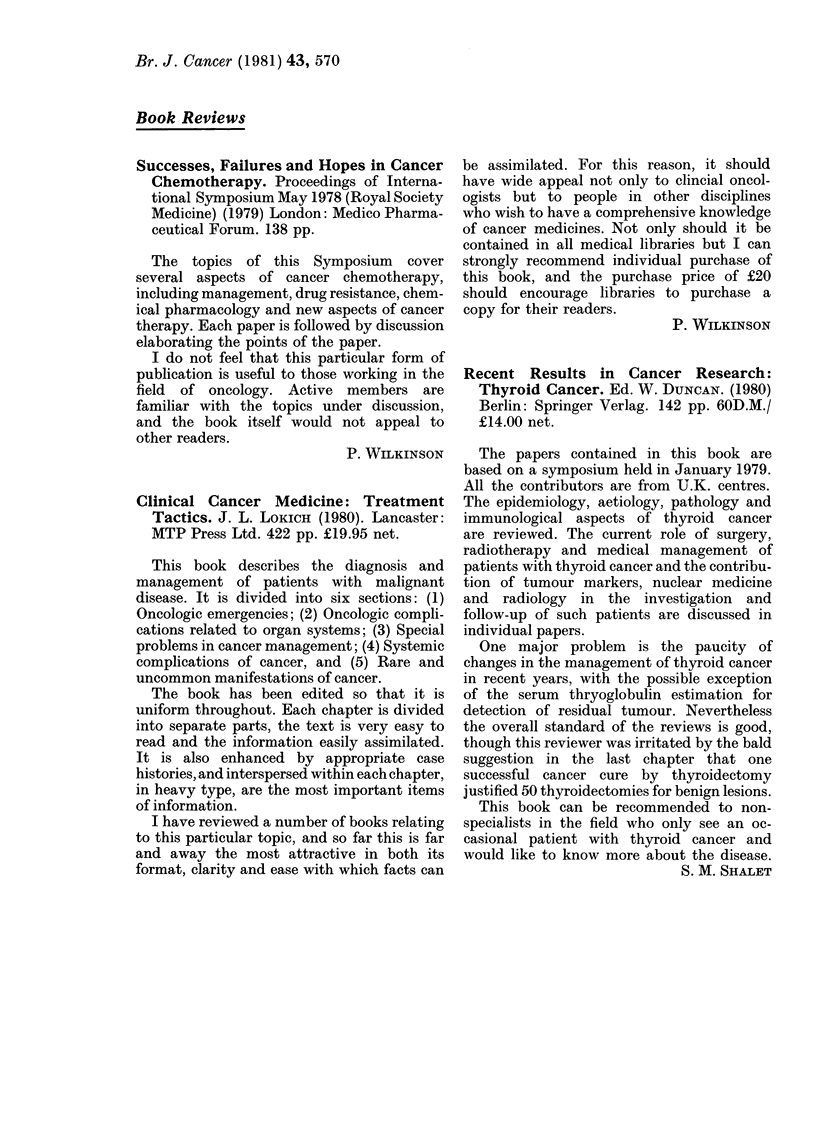# Recent Results in Cancer Research: Thyroid Cancer

**Published:** 1981-04

**Authors:** S. M. Shalet


**Recent Results in Cancer Research: Thyroid Cancer.** Ed. W. DUNCAN. (1980) Berlin: Springer Verlag. 142 pp. 60D.M./ £14.00 net.

The papers contained in this book are based on a symposium held in January 1979. All the contributors are from U.K. centres. The epidemiology, aetiology, pathology and immunological aspects of thyroid cancer are reviewed. The current role of surgery, radiotherapy and medical management of patients with thyroid cancer and the contribution of tumour markers, nuclear medicine and radiology in the investigation and follow-up of such patients are discussed in individual papers.

One major problem is the paucity of changes in the management of thyroid cancer in recent years, with the possible exception of the serum thryoglobulin estimation for detection of residual tumour. Nevertheless the overall standard of the reviews is good, though this reviewer was irritated by the bald suggestion in the last chapter that one successful cancer cure by thyroidectomy justified 50 thyroidectomies for benign lesions.

This book can be recommended to nonspecialists in the field who only see an occasional patient with thyroid cancer and would like to know more about the disease.

S. M. SHALET